# Progress in Dermatology

**Published:** 1903-09-19

**Authors:** 


### Progress in Dermatology.

(*Concluded from page* 419.)

Syphilis.—From the enormous material of 30,000 cases of syphilis in the province of Castamuni, Professor V. Düring [6] gives a very interesting summary of his general impressions. The disease here is endemic and not sporadic, which makes matters different from the ordinary conditions. For instance, accidental infection was more common than venereal, chancre being very rare (not 4 in 1,000), and the scar of the primary lesion was to be found in less than 100 cases in all. This primary lesion was never to be found in children, though the subjects of acquired syphilis. In previous reports of endemic syphilis the proportion of tertiaries to secondaries has been 2 to 1. This according to Von Düring does not tally with his facts. His figures are nearer 1 to 1·2, and he thinks that corrected they would work out nearer to 1 to 2. Even so they are largely in excess of the European statistics which give 1 to 10. Endemic syphilis differs from the malignant variety. The increase in the number of tertiaries, their precocity, extension, gravity and long duration is due to want of treatment. Disease of the palate, pharynx and nose were very frequent, while affections of the eye and nervous system were remarkably rare. Neurasthenia and melancholy he does not admit; and he only saw three cases of tabes in the town and not in the country, and only in one case was there a history of syphilis. He thinks that the chronic intermittent treatment by mercury of Fournier is dangerous in that it is responsible for nervous disorders. Von Düring admits that the "toxine theory of tertiarism is in direct opposition to certain clinical facts." He holds that the "value and consequence of Profeta's law is equal to nil." The dangers of infection by congenital syphilis, as depicted by French authors, must be considerably exaggerated. With regard to late hereditary syphilis, affections of the joints, especially the knee-joint, occurred with remarkable frequency, while interstitial keratitis was exceedingly rare. This is quite the reverse with us. Superficial diffuse glossitis was seen very frequently and if combined with other changes, it would warrant the diagnosis of congenital syphilis. This is not the same as what is known as geographical tongue. E. H. Douty [7] holds that the "open-air treatment" is as useful in early syphilis as in tuberculosis. The stimulation of the light, air, and bracing climate upon the metabolism is frequently of the utmost importance in syphilitics who are not in the best bodily condition to resist the disease. Mercury he holds is not an antidote to the syphilitic poison; it helps the tissues of the patient in the fight against the disease. He has observed that the man who throws off the disease with least trouble is the well-fed sportsman, who, living an out-of-door life with plenty of good food, may take his mercury irregularly, yet is hardly troubled at all by his complaint. On the other hand, the student who lives poorly may be most careful in his medicine-taking, yet has a hard fight for it. Tuberculosis is very common in syphilitics. The proportion among the male patients at high-latitude phthisis sanatorial resorts who have had syphilis is very high—30 to 50 per cent. Of course, alcohol ruins a man's resistance; but there is room for the consideration as to whether, if these men had not been treated by open-air, rest, forced feeding, and graduated exercise in a bracing and healthy climate, they would never have succumbed to phthisis and would have early lost their syphilis. It is an open question with him as to whether phthisis will ever be eliminated from the community without this treatment of syphilis. Pruritus in syphilis is generally supposed to be of very rare occurrence, yet Louis Regis [8] has collected several instances, and holds that it is not so uncommon as is supposed. Many forms of syphilides are accompanied by tingling and itching in more or less marked degree. Other complaints may be superadded, however, such as seborrhœa, which call for several treatments. Dr. Steuber [9] raises again the question of frambœsia tropica and its distinction from syphilis as occurring among the natives of German East Africa. In frambœsia there is at first a general eruption about the body, including the scalp, consisting of small, intensely itching papules. These papules become slightly raised, and light grey pin-head sized points appear which become seropurulent and break down to form dirty grey crusts. When these fall off or are removed, a granulating ulcer is left which rises above the surrounding edges giving rise to the frambœsial lesion. The two diseases are distinct from one another, and are recognised severally by natives, who call them by different names. They have besides undoubtedly occurred on the same patient. Drs. Galloway and Macleod [10] give notes of two cases which resembled one another very nearly and in both of which the diagnosis was very difficult. One was a case of erythema multiforme, resembling lupus erythematosus, with tubular nephritis, and the other one of lupus erythematosus resembling erythema multiforme, with alcoholism and hepatic cirrhosis. In both cases there was evidence of toxæmia of a severe degree. In one the condition was one of a rapid action of a virulent toxin circulating in the blood and causing an acute inflammatory disturbance in the skin, leading to a fatal termination in a few months; while in the other the toxin was much less virulent, its action was prolonged for years, and set up a more chronic type of dermatitis. As the authors say, it were foolish to seek for another powerful factor, especially one so obscure and uncertain in its effects as some remotely acting consequence of tuberculosis, as has been suggested, when we have evidence before us of such profound toxæmia. This contention of theirs, they point out, is on all fours with Dr. Wilfrid Warde's contention that the absorption of pyogenetic toxin may result in one of the forms of toxæmia concerned in the production of lupus erythematosus. This latter observer[11] claims that "Lupus erythematosus is not a distinct pathological entity, but merely one instance of a common process frequently met with in a certain class of individuals, atrophic rhinitis being another." He holds that the essential symptom is a pernicious œdema due to a paralysis and dilatation of small blood-vessels, which tends to complete destruction, together with the granulation tissue formed with a view to repair. This œdema and vascular degeneration depend (*a*) indirectly on a feeble circulation with malnutrition of the vessel walls, or strains on the vessels through flushing and on anatomical conditions (stretching of skin, etc.). (*b*) Directly on any cause capable of producing a surface œdema, *e.g.*, exposure to heat, cold, burns, and the presence on the skin of various efflorescences due to poisons, certain fevers, microbic activity, etc. The condition of skin termed lupus erythematosus is "merely a stage in the course of many different affections—a step in the pathological ladder by which a damaged part, unable to achieve its own repair, is destroyed and replaced by fibrous tissue. In a certain class of individuals the repair is indefinitely prolonged and may appear to be altogether postponed, and then the condition becomes known as lupus erythematosus." In the treatment of lupus erythematosus, Regner[12] speaks favourably of the high frequency currents, stating that this method is more rapid than phototherapy. Norman Walker[13] explains that a mistake has been made in applying the light to lupus erythematosus with the use of pressure. He had observed how that one patient was always better after fishing for the glare from the water affected the disease favourably. He now directs the light from a London hospital lamp at about 12 inches distance and has had good results. He also finds that 1 in 4,000 adrenalin chloride solution aids the cure. In his Lettsomian lectures Dr. Radcliffe Crocker[14] discusses the treatment of dermatitis. A large proportion of inflammatory diseases of the skin are of compound origin, bacteria often being found in combination, and the disease takes on different characters according to the nature of the combination. Intestinal conditions leading to the absorption of visceral toxins and ptomaines lead to many forms of eruptions, and this may explain the connection of gout and skin eruptions. The cerebral nervous system acts chiefly as a controlling influence over the sympathetic nerve system in regard to the intensity of the eruption. The central system controls the extent but not the nature of the eruption as a rule; the character being due to individual peculiarities, etc. He uses arsenic in psoriasis, lichen planus, and in the worst forms of lichen acuminatus; in erythema, especially E. hæmorrhagicum, chronic urticaria not dependent upon digestive disturbances. Arsenic should be long continued in these cases. In recurring sweat eruptions, in pemphigus and dermatitis herpetiformis, though not specific it often does good, and is useful in preventing bromide and iodide eruptions. It is useless in eczema and in diseases associated with gastric disturbances, *e.g.*, acne rosacea, urticaria of gastric origin, etc. Not good in evoluting stage of psoriasis, nor when patches are congested. He thinks sodium cacodylate a failure in skin diseases. Salicin has advantages over arsenic, and covers much the same ground. It is most useful in diseases which are probably of microbic origin, *e.g.* psoriasis, lichen planus (especially acute), pityriasis rosea. Not useful in psoriasis of scalp. Salicin is less likely to disagree than the salicylates. Thyroid extract is useful in prurigo, lichen acuminatus, and ichthyotic eczema; in lupus vulgaris it must be continued for a long time.

[6] Brit. Jour. of Dermatol., Jan. [7] Brit. Med. Jour., Feb. 28. [8] Ibid., July 11. [9] Ibid., March 28. [10] Brit Jour. of Derm., March. [11] Ibid., May. [12] Brit. Med. Jour., Feb. 7. [13] Ibid., June 27. [14] Ibid., March 21.

